# The Lifestyle Modifications and Endometrial Proteome Changes of Women With Polycystic Ovary Syndrome and Obesity

**DOI:** 10.3389/fendo.2022.888460

**Published:** 2022-06-22

**Authors:** D. Abdulkhalikova, A. Sustarsic, Eda Vrtačnik Bokal, N. Jancar, M. Jensterle, T. Burnik Papler

**Affiliations:** ^1^ Department of Human Reproduction, Division of Obstetrics and Gynaecology, University Medical Centre Ljubljana, Ljubljana, Slovenia; ^2^ Faculty of Sports, University of Ljubljana, Ljubljana, Slovenia; ^3^ Department of Endocrinology, Diabetes and Metabolic Diseases, Division of Internal Medicine, University Medical Centre Ljubljana, Ljubljana, Slovenia

**Keywords:** PCOS (polycystic ovary syndrome), obesity, endometrium, infertility, weight loss, proteome, glucose, testosterone

## Abstract

Polycystic ovary syndrome (PCOS) is a polyendocrine disorder and the most common endocrinopathy in women of reproductive age. Affected women have an elevated prevalence of being overweight and obese. Our study sought to determine how weight loss associated with lifestyle changes affects the endometrium specific proteome, endocrine-metabolic characteristics, and motor capabilities of obese women with PCOS and infertility. A group of 12 infertile women under the age of 38 with PCOS and BMI ≥30 kg/m^2^ were included in the study. An evaluation was performed by a gynecologist and an endocrinologist. The weight-loss program lasted 8 weeks under the guidance of a professional trainer. Endometrial sampling during a period of implantation window for proteome determination was performed before and after weight loss. In endometrial samples at the end of the study increased protein abundance was recorded for Legumain, Insulin-like growth factor-binding protein 7, Hepatocyte growth factor receptor, Keratin, type II cytoskeletal 7, and Cystatin-B, while the B-lymphocyte antigen CD20 protein abundance decreased. Our results also indicate significantly lowered fasting blood glucose level and free testosterone concentration and significant improvements in body composition and physical capacity. This study may open up the venues for investigating important biomarkers that may affect endometrial receptivity.

**Clinical Trial Registration:**

https://clinicaltrials.gov/ct2/show/NCT04989244?term=NCT04989244&draw=2&rank=1, identifier: NCT04989244.

## Introduction

Polycystic ovary syndrome (PCOS) encompasses a large group of endocrine disorders that arise due to diverse pathogenetic mechanisms but are combined according to the principle of similar clinical symptoms. PCOS is the main reason for ovulation disfunction and the most important complex endocrine disorder of women of reproductive age. The prevalence of PCOS is between 6 and 10% according to The National Institute of Health criteria and up to 15% according to the broader Rotterdam criteria  (1).

In general, PCOS patients have a significantly higher incidence of overweight, obesity, and central obesity (2). Obesity exacerbates the pathophysiological events of PCOS and is associated with inferior reproductive, endocrine, metabolic, and sociological functioning. The estimated prevalence of overweight and obese PCOS patients in different populations of women varies. According to rough estimates, obesity is present in 30 to 70% of patients (3).

Insulin resistance is considered the main pathophysiological feature in the development of PCOS, leading to multiple defects in the regulation of carbohydrate, fat, and purine metabolism. Obesity, especially the abdominal type, has a strong relationship to insulin resistance and hyperinsulinemia (4). Women, affected by PCOS have an increased prevalence of several comorbidities, which, in addition to obesity, include hypertension, metabolic syndrome, dyslipidemia, cardiovascular diseases, and type 2 diabetes mellitus. Insulin resistance is accompanied by reactive hyperinsulinemia, which over activates androgen-synthesizing cells of the ovary and adrenal cortex. Obesity and hyperinsulinemia have been associated with low levels of sex hormone-binding globulin (SHBG) (5). This further increases the concentration of biologically available circulating androgens, which dysregulates folliculogenesis and leads to the sequence of pathological events in PCOS patients (4, 6).

PCOS is the commonest cause (70%) of anovulatory subfertility (7). Despite good treatment options for anovulation, pregnancy rates in women with PCOS are lower and miscarriages are more common. Both obesity and PCOS are associated with a low rate of spontaneous pregnancy and a lower rate of conception in IVF procedures (8–10). Excessive body weight may trigger mechanisms that negatively affect several processes - ovulation, oocyte maturation, endometrial receptivity, implantation, and pregnancy continuation. Pregnant women with PCOS and obesity have higher rates of miscarriage and pregnancy complications and lower live births rates (5, 8, 11, 12). Extensive evidence suggests that weight reduction can improve health and fertility issues in obese women with PCOS. A weight loss of even 5–10% leads to improvement in endocrine-metabolic characteristics, menstrual cyclicity, and fertility (13, 14). The treatment of choice for overweight and obese women with PCOS is, therefore, weight loss through lifestyle modification (15).

The endometrial proteome is a very complex combination of different proteins. Many of them have already been described in the context of endometrial receptivity (16–19). Several different proteins were discovered lately and have been presented as endometrial receptivity related factors, but the number and profile of these proteins differed substantionally among the studies (20).

PCOS patients apperently have altered endometrial receptivity for implantation (21). All these biological processes and molecular mechanisms are largely controlled by proteins. Therefore, knowing the protein profile can provide us an insight into these complex events (19).

Some evidence suggests that the functioning of the endometrium is also compromised by obesity. Body mass index (BMI) increase leads to a significant decrease in embryo implantation rate, pregnancy rate, and live birth rate (10, 22, 23). The true impact of body weight on oocytes, embryos, and endometrium is not fully understood, however, the uterine environment appears to play a key role (10). Endometrial gene expression in the window of implantation is altered in obese women and is more pronounced when infertility and PCOS are associated (24). Weight loss improves the reproductive function of women with PCOS, but the exact mechanism of the impact of weight loss on endometrial receptivity has not been clarified.

Given the lack of knowledge regarding the relationship between PCOS, obesity, and changes of the endometrium specific proteome, our study aimed to identify the differentially abundant proteins as potential diagnostic and predictive biomarkers and to compare the endometrium protein profiles before and after the weight loss and lifestyle modifications.

We also sought to determine how weight loss associated with lifestyle changes (balanced and diverse diets, reduced alcohol and tobacco consumption, regular exercise, stress management, positive self-esteem, etc.) affect motor capabilities, endocrine-metabolic characteristics, and the regulation of the menstrual cycle of obese women with primary and secondary infertility.

## Materials and Methods

### Study Population

Our prospective case-crossover open-label study has been carried out by the Department of Human Reproduction, Division of Obstetrics and Gynecology in collaboration with the Department of Endocrinology, Diabetes, and Metabolic Diseases, Division of Internal Medicine of the University Medical Centre Ljubljana and the Faculty of Sport, University of Ljubljana, Slovenia from September 2017 to June 2018. A total of 52 consecutive obese PCOS patients with primary or secondary infertility were invited to participate in the study.

Inclusion criteria:

1. Age ≤38 years,
2. BMI ≥30 kg/m^2^,
3. Primary or secondary infertility,
4. PCOS, phenotype A, diagnosed according to revised Rotterdam criteria (25), characterized by concomitant presence of clinical or biochemical hyperandrogenism, ovulatory dysfunction and polycystic ovarian morphology (26).

Exclusion criteria:

1. Use of hormonal contraception, intrauterine device, hormone replacement therapy, metformin or myo-inositol in the last 3 months,
2. Distance and unreliable cooperation.

**Figure 1:**
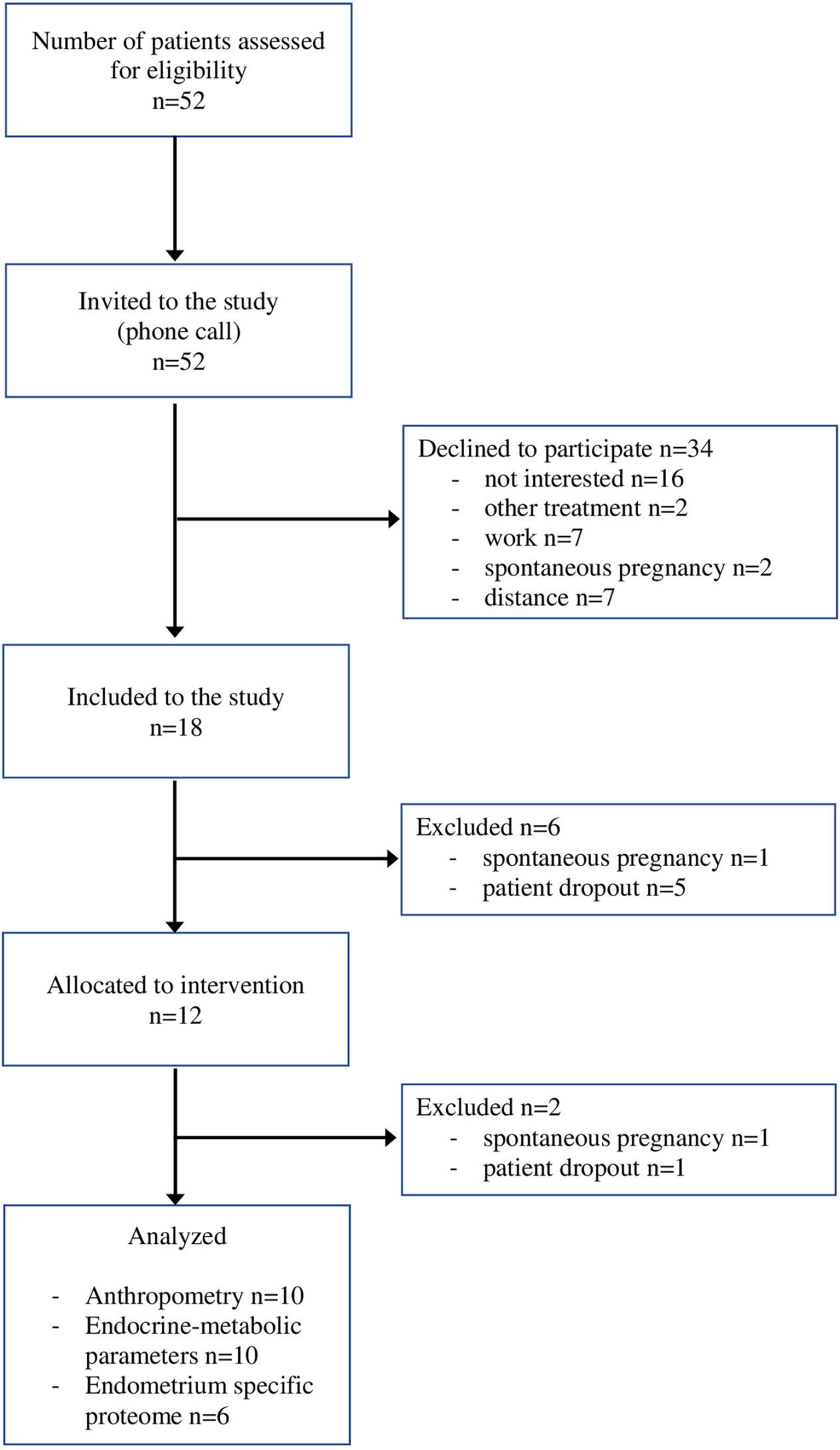
The flow chart of patient recruitment.

The final study group consisted of 12 infertile women who met the inclusion criteria stated above (**Figure 1**).

### Study Design

All participants were examined before the beginning of the study. A standard fertility evaluation and examination of both partners were performed by a gynecologist. To determine endometrium specific proteome, endometrial sampling during a period of implantation window (21st-23rd day of the menstrual cycle) was performed in the outpatient setting without anesthesia and cervical canal dilatation, using a single-use endometrial suction curette (Rampitella Ri.Mos.S.R.L. Mirandola, Italy). Endometrial specimens were stored at -70°C.

This was followed by a clinical examination by an endocrinologist with the evaluation of body characteristics (BMI, body weight, body height, level of visceral fat) and endocrine-metabolic parameters. This included: hemodynamic parameters (systolic and diastolic blood pressure, heart rate), metabolic markers (homeostatic insulin resistance model (HOMA-IR), fasting and postprandial plasma glucose and insulin), hormones and proteins (sex hormone binding globulin (SHBG), free testosterone, free androgen index (FAI), androstenedione, dehydroepiandrosterone (DHEA), follicle stimulating hormone (FSH), luteinizing hormone (LH), and anti-Müllerian hormone (AMH). Androstenedione was measured by specific double antibody RIA using 125 I-labeled hormones (Diagnostic Systems Laboratories, Webster, Tx). Total and free testosterone levels were measured by coated tube RIA (DiaSorin, S. p. A, Salluggia, Italy and Diagnostic Products Corporation, LA, respectively). LH and FSH were measured using an immunometric assay (Diagnostic Products Corporation, LA). Intra-assay coefficient of variation (CV) for androstenedione ranges from 5.0 to 7.5%, inter-assay CV from 4.1 to 11.3%; for free testosterone, intra-assay CV is 7.7–19.3% and inter-assay CV 6.4–13.2%; for total testosterone, intra-assay CV is 5.1–16.3% and inter-assay CV 7.2–24.3%. Intra-assay CV for SHBG is 2.5–5.3% and inter-assay CV 4–6.6%.

The standard 75-g oral glucose tolerance test (OGTT) was performed (27). Glucose levels were determined using the standard glucose oxidase method (Beckman Coulter Glucose Analyzer, Beckman Coulter Inc., CA, USA). Insulin was determined by immunoradiometric assay (Biosource Europe S.A., Nivelles, Belgium). Insulin resistance (IR) was calculated by the homeostasis model assessment for IR (HOMA-IR): fasting serum insulin (mU/l) x fasting plasma glucose (mmol/l)/22.5, cut off>2.5 (28). Impaired glucose tolerance was identified by 2-hour glucose levels between 7.8 and 11.0 mmol/l, as defined by the American Diabetes Association criteria (27).

Anthropometric measurements were assessed at the Faculty of Sport. The patients completed the questionnaire on overall well-being, stress, sports activities, lifestyle, and nutrition habits. The body characteristics were measured with a Tanita model BC 601, which calculates body composition using bio-electric impedance analysis. The motor capability assessment consisted of 9 tests, which were used to measure flexibility, strength, and endurance. In addition, we measured abdominal, waistline, and thigh circumferences as well. Waist circumference was measured in a standing position midway between the lower costal margin and the iliac crest. BMI was calculated as the weight in kilograms divided by height in meters squared.

At the beginning of the study, a healthy lifestyle was actively promoted in the group by a fertility specialist and personal trainer, with a detailed conversation and a leaflet with all the information about the dietary recommendations. A reduced calorie diet consisting of carbohydrates preferably with a low glycemic index was advised. The participants were encouraged to increase their consumption of fibers, whole grains, cereals, fruits, and vegetables. Under the supervision of a professional trainer, the eight-week weight-loss program included sixteen exercise units, and was held twice a week at the Faculty of Sport. Furthermore, aerobic activity of at least 30 minutes per day (cycling, speed walking, swimming, etc.) in the home environment was strongly recommended.

The Subjects Were Re-Examined by a Gynecologist, an Endocrinologist, and a Personal Trainer After They Completed the Weight Loss Program. Endometrial Samples Were Also Collected.

### Protein Microarray Analysis

Twelve endometrial samples taken from six patients were analyzed at Sciomics GmbH (Heidelberg, Germany) to identify protein differences in tissue samples before and after the weight loss. Proteins were extracted with scioExtract buffer (Sciomics). The bulk protein concentration was determined by BCA assay. The protein concentration was adjusted for one hour with scioDye 1 and scioDye 2. After two hours the reaction was stopped, and the buffer was exchanged for PBS. All samples were stored at -20°C until use. The 12 samples were analyzed in a dual-color approach using a reference-based design on 12 scioDiscover antibody microarrays (Sciomics) targeting 1360 different proteins with 1830 antibodies. Each antibody is represented on the array in four replicates. The arrays were blocked with scioBlock (Sciomics) on a Hybstation 4800 (Tecan, Austria) and afterwards the samples were incubated competitively using a dual-color approach. After incubation for three hours, the slides were thoroughly washed with 1x PBSTT, rinsed with 0.1x PBS as well as with water, and subsequently dried with nitrogen.

### Statistical Analysis

The results are presented as means ± SD or percentages as appropriate. The differences between the start and end state of the menstrual cycle were analyzed using the chi-square test. Normal data distribution was checked with the Shapiro–Wilk test. The t-test was used to compare the before and after parameters for the normally distributed data (paired samples t-test). Non-normally distributed data were tested with Mann-Whitney U and Wilcoxon signed ranks tests. A p-value of ≤ 0.05 was considered statistically significant. Statistical analysis was performed using IBM SPSS Statistics, version 21 (IBM Corp, Armonk, NY).

Endometrial sample analyses: slide scanning was conducted using a Powerscanner (Tecan, Austria) with identical instrument laser power and adjusted PMT settings. Spot segmentation was performed with GenePix Pro 6.0 (Molecular Devices, Union City, CA, USA). Acquired raw data were analyzed using the linear models for microarray data (LIMMA) package of R-Bioconductor after uploading the median signal intensities. A specialized invariant Lowess method was used for normalization.For samples analysis, a one-factorial linear model was fitted with LIMMA resulting in a two-sided t-test or F-test based on moderated statistics. All presented p-values were adjusted for multiple testing by controlling the false discovery rate according to Benjamini and Hochberg. Proteins were defined as differential for |logFC| > 0.5 and an adjusted p-value < 0.05. Differences in protein abundance between different samples or sample groups are presented as log-fold changes (logFC) calculated for the basis 2. In a study comparing samples versus control a logFC = 1 means that the sample group had on average a 2^1^ = 2-fold higher signal than the control group. logFC = −1 stands for 2^−1^ = 1/2 of the signal in the sample as compared to the control group.

## Results

### The Lifestyle Modifications Affect the Endometrial Proteome of Women With Polycystic Ovarian Syndrome and Obesity

Between END samples (final visit) and START samples (baseline visit), an increased protein abundance was recorded for Legumain, Insulin-like growth factor-binding protein 7 (IGFBP-7), Hepatocyte growth factor receptor (HGF receptor), Keratin, type II cytoskeletal 7 (CK-7), and Cystatin-B, while the B-lymphocyte antigen CD20 (CD20) protein abundance decreased.

**Figure 2:**
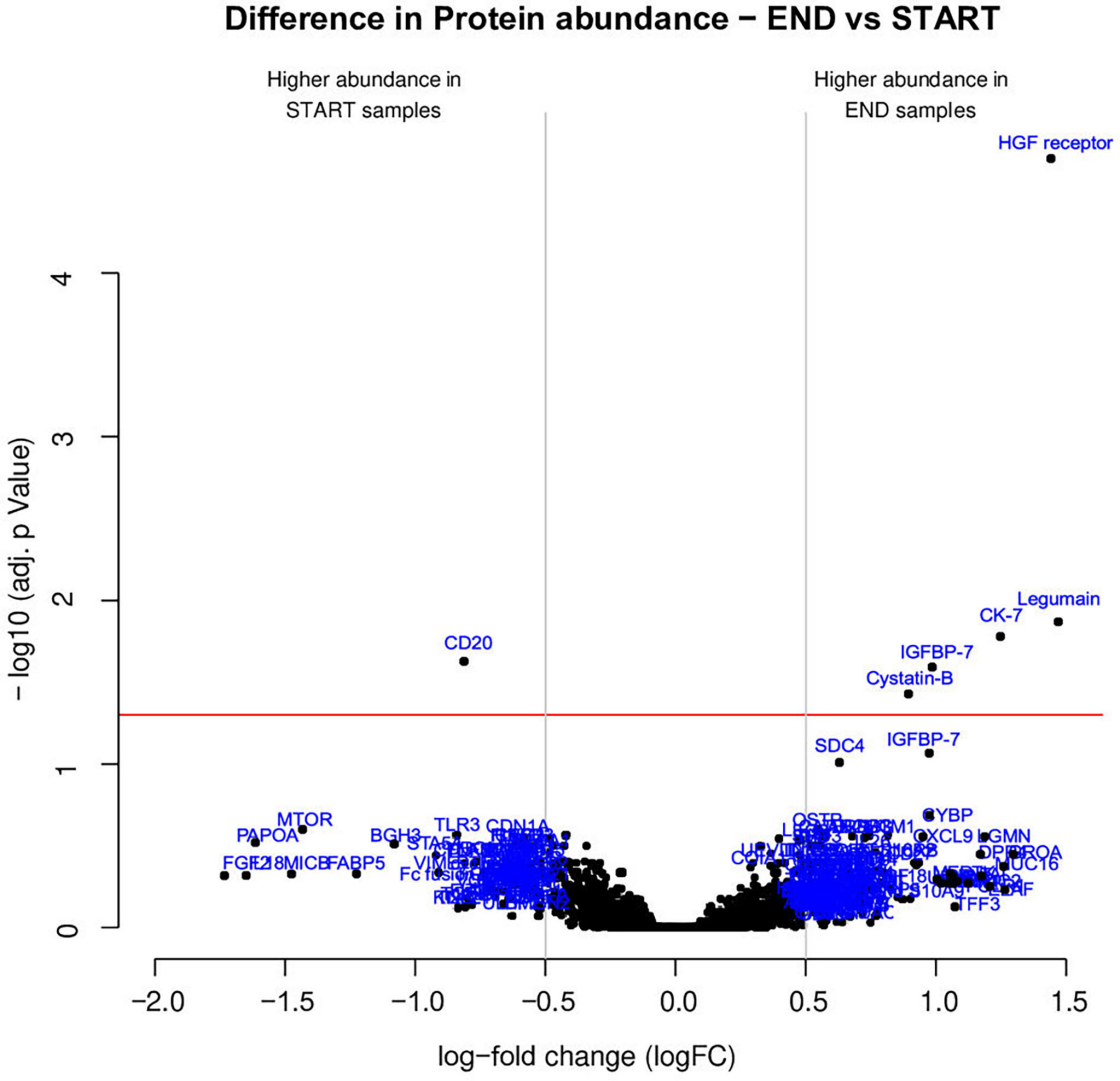
Several proteins exhibited distinct abundance variations in END samples (final visit) and START samples (baseline visit). The volcano plot visualizes the p-values (adjusted for multiple testing) and corresponding log-fold changes (logFC). A significance level of adj. p-value = 0.05 is indicated as a horizontal red line. The logFC cutoffs are indicated as vertical lines. Proteins with a positive logFC had a higher abundance in END samples, proteins with a negative value in START samples.

**Figure 3:**
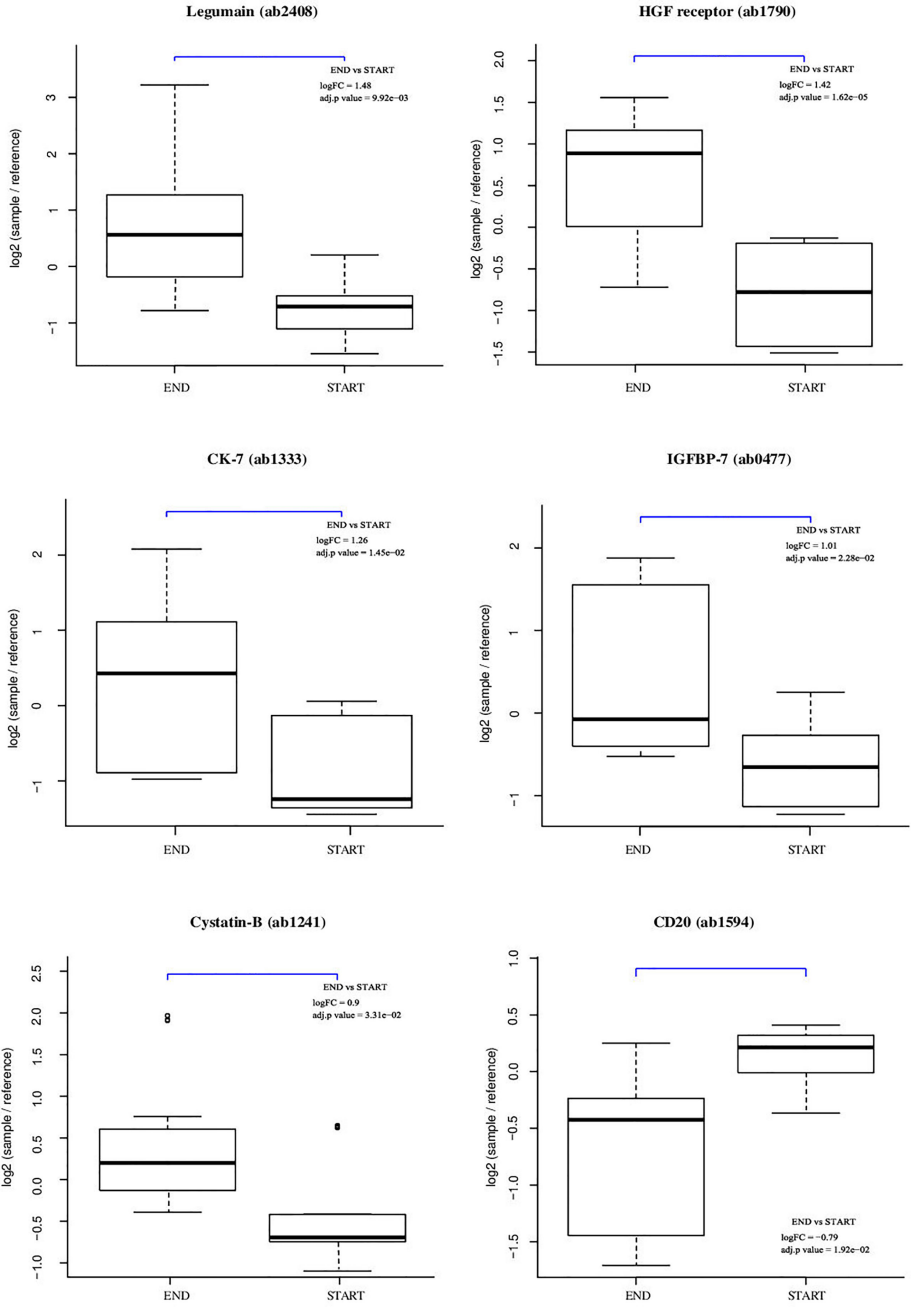
Individual array values for a set of differential proteins. Each sample is measured by four replicate spots per array.

The results of the statistical analysis are summarized in the volcano plot (**Figure 2**) and listed in **Table 1**. Relative protein levels are presented in **Figure 3**. Furthermore, **Supplementary Table S1** lists differential proteins including those that do not reach the logFC and significance thresholds simultaneously.

**Table 1:** Proteins with differential abundance in END samples (final visit) and START samples (baseline visit).

Protein	AntibodyID	logFC	AveExp	adj.p-val	Uniprot-Entry
Legumain	ab2408	1.48	10.95	9.9e-03	Q99538
HGF receptor	ab1790	1.42	12.91	1.6e-05	P08581
CK-7	ab1333	1.26	13.63	1.4e-02	P08729
IGFBP-7	ab0477	1.01	13.40	2.3e-02	Q16270
Cystatin-B	ab1241	0.90	13.40	3.3e-02	P04080
CD20	ab1594	-0.79	14.37	1.9e-02	P11836

logFC – log fold changes (positive logFC value – higher abundance in END samples, negative logFC value – higher abundance in START samples); adj.p-val – p-values adjusted for multiple testing; Uniprot-Entry – the Uniprot-Identifier.

### Lifestyle Modifications Reduced the Levels of Fasting Glucose and Free Testosterone and Positively Affected Body Characteristics and Motor Capability Skills

Most women improved their eating habits after the interventions, have started regular exercises, slept longer. At the end of the study, there was a statistically significant improvement in balanced food consumption (p <0.001), regular exercise (p <0.001), socializing with family and friends (p = 0.031), and changes to a positive attitude (p = 0.004).

#### Hemodynamic Parameters

**Table 2** shows the systolic blood pressure drop by 8.500 mmHg (p=0.068), with no changes in the diastolic blood pressure (p=0.140) and heart rate (p=0.278).

**Table 2:** Comparison between baseline and final measurements of hemodynamics.

	Measurements	µ	σ	ST. N. µ	Δ	Δ%	t	p
Systolic blood pressure (mmHg)	Baseline	127.6	20.9	6.6	8.5	6.6%	2.1	0.068
	Final	119.1	17.2	5.4				
Diastolic blood pressure (mmHg)	Baseline	83.8	15.2	4.8	6.6	7.8%	1.6	0.140
	Final	77.2	8.7	2.8				
Heart rate (bpm)	Baseline	82.1	8.6	2.7	4.0	4.8%	1.2	0.278
	Final	78.1	9.4	2.9				

Legend: µ – average; σ – standard deviation; ST. N. µ – standard error of average; Δ – absolute change; Δ% – absolute change in percentage; t – t-test statistics; p – statistical significance.

#### Metabolic Parameters

**Table 3** shows a statistically significant decrease in fasting glucose concentration by 0.380 mmol/l (p=0.040). The data regarding HOMA-IR (p=0.053) and fasting insulin concentration (p=0.065) revealed a marginal trend toward significance.

**Table 3:** Comparison between baseline and final measurements of metabolic markers.

	Measurements	µ	σ	ST. N. µ	Δ	Δ%	T	p
HOMA-IR	Baseline	4.9	2.6	0.8	2.0	40.8%	2.3	0.053
	Final	2.9	1.1	0.3				
Fasting glucose (mmol/l)	Baseline	5.7	0.5	0.2	0.4	7%	2.4	0.040
	Final	5.3	0.3	0.1				
Fasting insulin (pmol/l)	Baseline	18.9	9.3	2.9	6.8	36%	2.1	0.065
	Final	12.1	4.3	1.4				

µ – average; σ – standard deviation; ST. N. µ – standard error of average; Δ – absolute change; Δ% – absolute change in percentage; t – t-test statistics; p – statistical significance. HOMA-IR – homeostatic model of insulin resistance.

#### Endocrine Parameters

There was a statistically significant decrease in free testosterone concentration by 3.777 pmol/l (p=0.002). SHBG concentration increased, although statistically insignificant, by 4.470 nmol/l (p=0.074). A positive trend was also observed in anti-Müllerian hormone concentration by 1.053 mg/l (p=0.093), though statistically insignificant. The average values of FAI index (p=0.637) and androstenedione concentration (p=0.218) did not change (**Table 4**).

**Table 4:** Comparison between baseline and final measurements of hormone and proteins.

	Measurements	µ	σ	ST. N. µ	Δ	Δ%	T	p
SHBG (nmol/l)	Baseline	30.0	14.3	4.5	-4.7	15.6%	-2.0	0.074
	Final	34.7	18.4	5.8				
Free testosterone (pmol/l)	Baseline	9.1	3.8	1.2	3.8	41.7%	4.3	0.002
	Final	5.3	1.9	0.6				
FAI index	Baseline	2.6	2.9	0.9	0.3	11.5%	0.5	0.637
	Final	2.3	1.6	0.5				
Androstendione (nmol/l)	Baseline	4.9	1.6	0.5	0.6	12.2%	1.3	0.218
	Final	4.3	1.4	0.4				
Anti-Müllerian hormone(mg/l)	Baseline	3.7	1.9	0.6	-1.1	29.7%	-1.9	0.093
	Final	4.8	2.9	0.9				

µ – average; σ – standard deviation; ST. N. µ – standard error of average; Δ – absolute change; Δ% – absolute change in percentage; t – t-test statistics; p – statistical significance. SHBG – sex hormone-binding globulin, FAI index-free androgen index.

#### Menstrual Cycle Regularity

**Table 5** shows a comparison between baseline and final reports of the menstrual cycle regularity. We have observed that the regularity of the menstrual cycle has improved, although this improvement was not statistically significant (p=0.073).

**Table 5:** Changes in the menstrual cycle.

		Baseline	Final	Total	p
Irregular	f	7	3	10	0.073
Regular	f	3	7	10	
Total	f	10	10	20	

p – statistical significance.

#### Body Characteristics

On average, 6.28 kg of body weight and 2.55% of body fat were lost, 1.85 kg of muscle mass were gained, BMI was reduced by 2.17 kg/m2, abdominal circumference by 6.31 cm, waistline circumference by 9.02 cm, and hip circumference by 6.31 cm (**Table 6**). All the results were statistically significant. More detailed information is available in **Table 6**.

**Table 6:** Comparison between baseline and final visit measurements of body characteristics and composition.

Parameter	Measurements	µ	σ	Δ	Δ%	t	p
Body weight (kg)	Baseline	110.2	14.7	-6.3	-5.7%	7.641	<0.001
	Final	103.9	14.2				
Body fat (%)	Baseline	44.6	5.3	-2.6	-5.7%	-3.181	<0.001
	Final	42.0	6.3				
Muscle mass (kg)	Baseline	54.5	3.9	1.9	3.4%	-4.07	0.003
	Final	56.4	4.8				
BMI (kg/m^2^)	Baseline	39.5	4.4	-2.2	-5.5%	6.701	<0.001
	Final	37.3	4.4				
Abdominal circumference (cm)	Baseline	121.1	8.0	-7.0	-5.2%	8.433	<0.001
	Final	114.1	7.8				
Waistline circumference (cm)	Baseline	109.7	9.9	-9.0	-8.2%	7.250	<0.001
	Final	100.7	9.8				
Hip circumference (cm)	Baseline	129.5	8.1	-6.3	-4.9%	8.433	<0.001
	Final	123.2	7.7				

µ – average; σ – standard deviation; Δ – absolute change; Δ% – absolute change in percentage; t – t-test statistics; p – statistical significance.

#### Motor Capabilities

There was a statistically significant improvement in all the parameters associated with motor capabilities (**Table 7**). Hip flexibility improved by 6.5 cm, the flexibility of the left shoulder by 3.57 cm, and of the right shoulder by 3.82 cm. After the weight-loss program, we noted an improvement in the number of achievable pull-ups (additional 2.27 repetitions), the strength of hip and back extensors (additional 53.23 seconds), achievable squats (19.23 additional repetitions), the maximum time of plank test (additional 37.23 seconds), and a lower heart rate during the 3-minute stepping test (11.62 beats per minute).

**Table 7:** Motor capability skills comparison during baseline and final visit measurements.

Parameter	Measurements	µ	σ	Δ	Δ%	t	p
Sit and reach test (cm)	Baseline	33	9	7	21%	-6.4	<0.001
	Final	40	8				
Left shoulder flexibility (cm)	Baseline	14	8	4	28%	5.4	<0.001
	Final	10	8				
Right shoulder flexibility (cm)	Baseline	18	7	4	22%	-3.2	<0.001
	Final	14	6				
Push-up (max. num.)	Baseline	15	5	12	80%	-7.9	<0.001
	Final	27	7				
Pull-up (max. num.)	Baseline	2	2	3	150%	-6.7	<0.001
	Final	5	3				
Sit-up (max. num. in 1 min)	Baseline	30	8	16	53%	-7.7	<0.001
	Final	46	10				
Lift legs on belly (max. s)	Baseline	143	79	54	38%	-4.5	0.001
	Final	197	88				
Squats (max. num.)	Baseline	41	11	20	49%	-10.7	<0.001
	Final	61	14				
Plank (max. s)	Baseline	60	16	37	62%	-5.4	<0.001
	Final	97	25				
3min stepping test (heart rate, 1 min)	Baseline	129	13	12	9%	5.0	<0.001
	Final	117	12				

µ – average; σ – standard deviation; Δ – absolute change; Δ% – absolute change in percentage; t – t-test statistics; p – statistical significance.

## Discussion

### Lifestyle Modifications Affect the Endometrial Proteome of Women With Polycystic Ovarian Syndrome and Obesity

As a result of our lifestyle interventions, an increase in protein abundance was observed for Legumain, IGFBP-7, HGF receptor, CK-7, and Cystatin-B while the CD20 protein abundance decreased.

There is growing evidence that the endometrium of PCOS patients has impaired functioning, which may correlate with a higher rate of implantation failure, higher risk of miscarriage, and adverse pregnancy outcomes (29–31). Alikhani et al. investigated the endometrium proteome in luteal phase of cycle in PCOS patients compared to healthy fertile individuals. The results of proteome analysis indicated significant imbalances in the endometrial tissue of women with PCOS (32). Amjadi et al. performed a comparison of the endometrium proteome between PCOS patients and control group. In that study, differences in expression of proteins, involved in oxidative stress, apoptosis, immunological response, inflammation, blood coagulation and cytoskeletal organization have been discovered (33). They found differentially expressed proteins involved in apoptosis, oxidative stress, inflammation, and immunological functions, as well as cytoskeletal organization and blood coagulation (33).

### Legumain

In our study we recorded an increased protein abundance for Legumain in endometrial samples at the end of lifestyle interventions, suggesting a positive effect of weight loss on endometrial receptivity.

Legumain is encoded by the *LGMN* gene. It is a cysteine endopeptidase that shows specificity for hydrolysis of asparaginyl bonds. Its activity was detected in the human placenta (34). Legumain has a role in the regulation of extracellular matrix remodeling (35). Bauersachs et al. investigated transcriptome profiles of endometrial samples from day 18 pregnant vs non-pregnant heifers. They identified Legumain as being up-regulated (preimplantation) in the endometrium (35). Ledgard et al. suggested that Legumain and tissue inhibitor of metalloproteinase 2 (TIMP2) regulate the trophoblast invasiveness and endometrial remodeling, essential for pregnancy (36).

Evans et al. established that Legumain is one of the key proteins localized to the epithelium of the receptive phase (mid-secretory) endometrium but largely absent from the non-receptive phase (proliferative) endometrium and the knockdown of this protein in epithelial cells functionally reduced their adhesive capacity (37).

According to newest findings of Bayramoglu et.al., Legumain expression correlates to grade of endometrial pathologic activity. In their very recent study, the authors investigated correlation of endometrial carcinoma to Legumain expression in human endometrium. Patients with higher Legumain expression were more likely to have higher carcinoma grade as well as carcinoma recurrence (38). Therefore, one could expect, that reducing the endometrial activity by losing weight and normalizing hormonal balance should result in down-regulation of Legumain expression in the endometrium, but our findings suggest just the opposite effect. It seems, that Legumain could not simply be used as a tumor marker of endometrial hyperproliferation in PCOS patients. On the other hand, Sun et al. reported, that a newly identified pseudogene of Legumain, derived from endometrial stroma and serum of endometriosis patients, could be a new predictive biomarker for ovarian endometriosis recurrence (39). One can only conclude that the action and regulation of Legumain expression in PCOS patients are still obscure and probably regulated specifically in certain physiological as well as pathological circumstances. Our findings could contribute to the understanding of the endometrial activity, hyperproliferation risk and implantation susceptibility.

**Figure 4:**
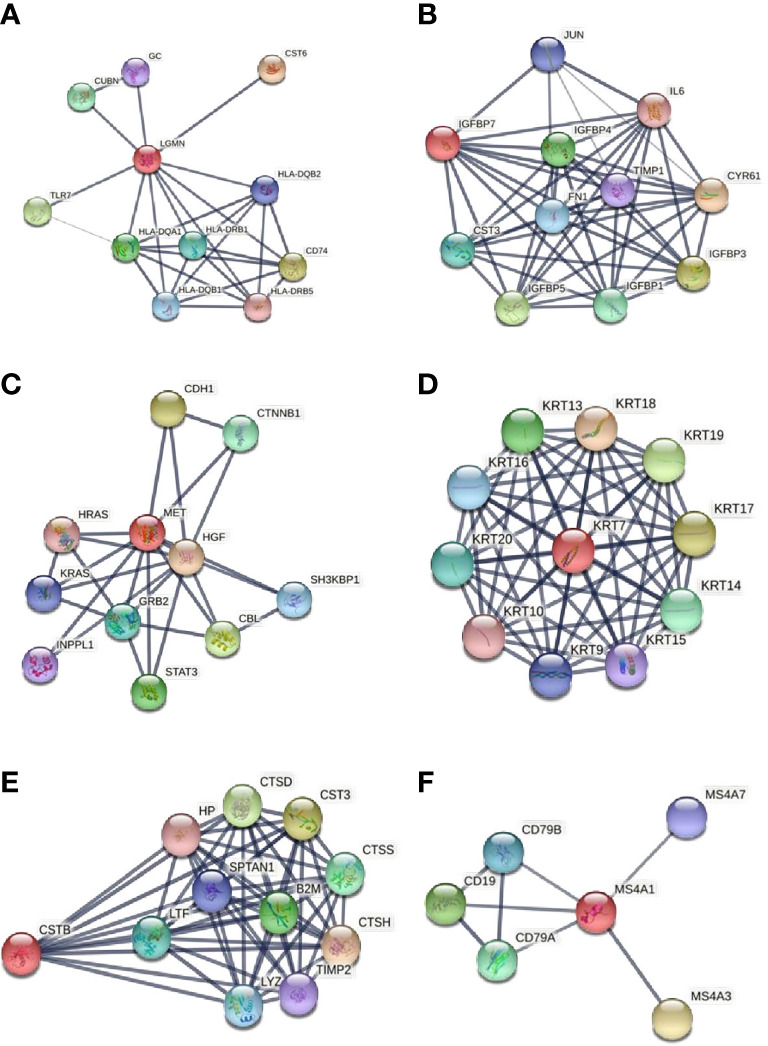
Functional associations of the studied proteins. The colored nodes signify a direct interaction with other proteins. The confidence cutoff for showing interaction links has been set to “high” (0.700) for CD20 and ‘highest’ (0.900) for Legumain, IGFBP-7, HGF receptor, CK-7, and Cystatin-B. **(A)** Legumain (*LGMN*), **(B)** IGFBP-7 (*IGFBP7*), **(C)** HGF receptor (*MET*), **(D)** CK-7 (*KRT7*), **(E)** Cystatin-B (*CSTB*), **(F)** CD20 (*MS4A1*). All abbreviations in figure 4 are explained in the **Supplementary File S2**.

Based on the available literature, Legumain does not appear to be linked with obesity. The functional associations of Legumain were explored using the STRING v11 clustering tool (**Figure 4**) (40).

There is a meaningful association between Legumain and Cubilin (CUBN), an endocytic receptor that plays a role in lipoprotein, vitamin, and iron metabolism. It is associated with the immune system process and embryonic development. The disordered expression of Cubilin in the early embryo may affect the conceptus–maternal interactions during the peri-implantation period in sheep (41). There is also a strong interaction between Legumain and vitamin D-binding protein (GC), which is involved in vitamin D storage and transport and possibly in the pathogenesis of idiopathic infertility (42). It is well known that vitamin D has a prominent impact on female reproduction functioning, including IVF outcome, PCOS, and endometriosis (43). Vitamin D deficiency might be involved in the pathogenesis of insulin resistance and metabolic syndrome in PCOS patients (44, 45). Results from an *in vivo* study showed that 1,25-Dihydroxyvitamin D3 supplementation induces the decidualization of rat endometrial cells (46). Legumain also appears to be involved in antigen processing and the presentation of exogenous peptide antigen *via* MHC class II, positive regulation of leukocyte chemotaxis, and positive regulation of immune system process.

### IGFBP-7

Our study recorded an increased protein abundance for IGFBP-7 in endometrial samples at the end of the lifestyle interventions, suggesting a positive effect of weight loss on endometrial receptivity.

IGFBP-7, also known as IGFBP-related protein 1, is a secreted glycoprotein, encoded by the *IGFBP7* gene. It originates from the IGFBP-related protein family, which binds insulin and insulin-like growth factor I (IGF-I). IGFBP-7 regulates the metabolism of IGF and affects the ability of IGF to bind the its receptor (47).

IGFBP-7 mRNA is expressed in different human tissues, its expression is also abundant in secretory endometrium. Tamura et al. investigated the ontology of IGFBP-7 mRNA in rat uterus during the peri-implantation period. They suggested IGFBP-7 might be important in preparing the uterus for successful implantation (47). Kutsukake et al. found out that IGFBP-7 is expressed in the endometrial stroma and glandular epithelium during the secretory phase of the menstrual cycle and plays an important role in decidualization (48). Domínguez et al. discovered that IGFBP-7 expression was present in both the epithelial and stromal fractions. IGFBP-7 mRNA levels in the late secretory stage were approximately 150-fold higher than in the proliferative stage, which led to the suggestion that IGFBP-7 is implicated in human endometrial receptivity (49). Liu et al. found that IGFBP-7 was mainly located in the glandular epithelium and the stroma and was upregulated after embryo implantation. The inhibition of IGFBP-7 induced a significant reduction in the number of implanted embryos and pregnancy rate (50).

There is also a relationship between IGFBP-7 concentration and obesity. Based on the study of IGFBP1-7 in obese pregnant women, women with gestational diabetes, and their fetuses, Lappas (51) found that preexisting maternal obesity and gestational diabetes are associated with lower levels of IGFBP-1 and IGFBP-7 in maternal and cord plasma.

The functional associations of IGFBP-7 were explored using the STRING v11 clustering tool (**Figure 4**) (40). IGFBP-7 is strongly associated with nine proteins involved in the regulation of IGF activity. The IGF-IGFBP system plays an important role in mediating fetal-maternal crosstalk, which is required for the establishment and maintenance of pregnancy (52). IGFBP-7 strongly interacts with interleukin 6 (IL6), a proinflammatory cytokine, produced by the human endometrium. It is known to be important in the endometrial function and embryo implantation. Endometrial levels of IL-6 peak at the time of implantation and are decreased in women with infertility and recurrent miscarriage (53). IL-6 is maximally expressed during the implantation window, both in the blastocyst and in the endometrium (54).

### HGF Receptor

Our study observed an increased protein abundance for the HGF receptor in endometrial samples at the end of the lifestyle interventions, suggesting a positive effect of weight loss on endometrial receptivity.

HGF receptor is encoded by the *MET* gene. It is a transmembrane type I tyrosine kinase receptor that transduces signals from the extracellular matrix into the cytoplasm by binding to hepatocyte growth factor (HGF). It regulates many physiological processes and is expressed in a variety of epithelial tissues, including the uterus end placenta (55).

In the ovine uterus, HGF mRNA expression was detected in stromal cells, whereas HGF receptor mRNA expression was noted in luminal and glandular epithelial cells. Chen et al. suggested that HGF could be crucial for uterine endometrium and trophectoderm/chorion function necessary for the establishment and maintenance of pregnancy (55).

Bauersachs et al. investigated transcriptome profiles of endometral samples from day 18 pregnant vs non-pregnant heifers. In this study, MET mRNA level was elevated in the endometrium of pregnant animals (35).

According to Stepanjuk et al., in a study of MUC20 expression in human endometrium, the expression of HGF receptor in the epithelium and HGF in the stroma increased in the receptive endometrium compared with the pre-receptive endometrium (56).

There is a connection between obesity and elevated serum HGF. HGF signaling is involved in the metabolic flux of glucose in various types of insulin-sensitive cells (57). HGF is expressed and secreted by human adipose tissue, its levels are elevated in overweight patients and raised with body mass index (58). This increase in serum HGF levels is significantly associated with the development of insulin resistance (59). On the other hand, there are some beneficial effects of raised HGF levels in obesity, as HGF considerably increases glucose metabolism and transport in myocytes and adipocytes (57).

The functional associations of HGF receptor (MET) were explored using the STRING v11 clustering tool (**Figure 4**) (40). HGF receptor shows a strong interaction with hepatocyte growth factor (HGF), transcription factor signal transducer and activator of transcription (STAT-3), and growth factor receptor-bound protein 2 (GRB2). Together they are implicated in the RAS guanyl nucleotide exchange factor activity. Previous studies have shown that the RAS gene family is mainly involved in the regulation of cell proliferation, differentiation, cell adhesion, and apoptosis (55). There is also an evident association between the HGF receptor and two proto-oncogenes of the RAS oncogene family - HRAS and KRAS. Both of them play an important role in the regulation of cell proliferation in response to growth factor stimulation. Studies on their roles in the female reproductive system are mainly focused on cancers, as they promote oncogenic events. Long et al. studied the role of KRAS in the endometrium of early pregnant mice; they found that KRAS can regulate the process of embryo implantation by regulating stromal cells’ proliferation and decidualization (60).

### CK-7

In our study, we recorded an increased protein abundance for CK-7 in endometrial samples at the end of the lifestyle interventions. To the best of our knowledge, CK-7 has not yet been associated with human endometrial receptivity. There is also no reliable connection between CK-7 and obesity in the reviewed literature.

Cytokeratins are keratin proteins of cytoskeletal intermediate filaments, found in different epithelia. There are 20 different cytokeratin isotypes in humans.

Cells of ovarian mesothelium, Fallopian tube, endometrium, and endocervix contain cytokeratin 7, 8, 18, and 19. Epitelia of the vagina and exocervix contain cytokeratins 4, 5, 6, 13, 14, 15, 16, and 19 (61). Cytokeratins are useful as markers of epithelial malignancies to reflect sustained cellular activity (62).

Keratin, type II cytoskeletal 7 also known as cytokeratin-7 (CK-7) is encoded by the *KRT7 *gene. It is abundantly expressed in the different epithelial tissues. CK-7 stimulates DNA synthesis in cells and is widely used as an epithelial marker. The immunostaining for CK-7 may help distinguish the Müllerian-subtype of mucinous gynecologic tumors from the lower gastrointestinal tract malignancies (63). According to some studies, CK-7 is highly expressed throughout the trophoblast lineage and serves as a marker for trophoblast cells (64). Monoclonal antibodies against CK-7 are a sensitive and reliable marker for the morphologic discrimination between invasive trophoblastic cells and decidual cells (65).

Riesewijk et al compared gene expression profiles of pre‐receptive versus receptive endometrium from the same fertile women. They found at least three fold CK-7 upregulation in receptive endometrium compared to pre-receptive endometrium (66).

The functional associations of CK-7 were explored using the STRING v11 clustering tool (**Figure 4**) (40). CK-7 shows strong interactions with other type I cytokeratins. Together they are involved in the pathway of keratinization. CK-18 is known as an endometrial epithelial cell-specific marker. CK-8 and CK-19 possibly cooperate in ensuring the normal development of placental tissues (67). There is an upregulation of a CK-13-containing intermediate filament network in luminal epithelial cells of the secretory phase and peri-implantation stage endometrium (68). The function of upregulation of CK-7 in the endometrium of PCOS patients after weight loss is a question that remains to be answered. Alterations in CK-7 expression in the endometrium are potentially related to the transformation of the intermediate filament cytoskeleton of epithelial cells.

### Cystatin-B

In our study, we recorded an increased protein abundance for Cystatin-B in endometrial samples at the end of the lifestyle interventions.

The Cystatin superfamily is involved in the inhibition and regulation of cysteine proteinases, neutrophil chemotaxis, tissue inflammation, and hormone processing (69).

Cystatin-B is an intracellular thiol proteinase inhibitor, encoded by the *CSTB* gene. It is widely expressed among different cell types and tissues. Like other members of the cystatin superfamily, Cystatin-B is a reversible and competitive inhibitor of cysteine proteases, cathepsin B, L, and S  (70).

The dysregulated expression of Cystatin-B appears to be associated with tumorigenesis (71, 72).

Forde et al. identified conceptus-derived proteins from pregnant heifers; they suggested Cystatin-B is one of the conceptus-derived proteins that may mediate conceptus-maternal interactions (73).

The reliable link between Cystatin-B and obesity is not yet clear. It has already been well established though that some cathepsins are elevated in obesity. Cathepsin B and S are overexpressed in adipose tissue of obese subjects. Taleb et al. observed a significant decrease in Cathepsin S enzymatic activity by 25% after weight loss (74). The role of Cystatin-B in adipose tissue remains to be explored.

The functional associations of Cystatin-B were explored using the STRING v11 clustering tool (**Figure 4**) (40). There is a strong association between Cystatin-B and cathepsin D, H, and S (CTSD, CTSH, CTSS) and with another inhibitor of cysteine proteinases cystatin C (CST3). Cathepsins derive from the family of lysosomal cysteine proteases. They are involved in many physiological processes, such as matrix molecule degradation and intracellular proteolysis (75). Cathepsins B, H, K, L, and S are required for the normal development and function of the human endometrium (76). Cystatin C is a major inhibitor of cathepsins B and L and is implicated in a biological process of regulation of tissue remodeling. There is some evidence that cathepsins B and L are necessary for normal embryo development and uterine decidualization (77). A recent study suggests that cathepsin S most likely supports embryonic implantation and that embryo has the ability to regulate the depth of its own invasion by expression of the cathepsin inhibitors (69). Cathepsins and cystatins expression was also demonstrated in human decidua and villi from patients with recurrent miscarriage. This suggests that the regulation of the cathepsin – cystatin system plays an important role in recurrent miscarriage. This indicates an important role of cathepsins and cystatins in recurrent pregnancy loss (75).

There is also a strong interaction of Cystatin-B with Metalloproteinase inhibitor 2 (TIMP2), which complexes with matrix metalloproteinases (MMPs) and irreversibly inactivates them. It is well known that the extravillous cytotrophoblasts achieve their invasive potential by producing high amounts of MMPs (78), which are capable of degrading components of the extracellular matrix. At the same time, the decidua secretes TIMPs to limit invasion. The expressive balance between MMPs and their tissue inhibitors plays a critical role in the balanced and limited invasion of trophoblast cells.

### CD20

Our study recorded a decreased protein abundance for CD20 in endometrial samples at the end of the lifestyle interventions. This may be associated with an improvement in the metabolic profile after weight loss and with a reduction in insulin resistance and glucose intolerance.

CD20 is a phosphorylated cell surface protein expressed exclusively by B lymphocytes. It is encoded by the MS4A1 gene. CD20 regulates Ca^2+^ transport across the plasma membrane. It is involved in the regulation of B cell activation and proliferation (79).

Lachapelle et al. found that the proportion of B lymphocytes (CD20+) was strikingly increased in endometria of habitual aborters (80). In the study of the role of CD20 in ovarian cancer prognosis Milne et al. proposed it as a positive prognostic factor in high-grade serous epithelial ovarian cancer (81).

Linkov et al. investigated changes in immune markers in the endometrium of morbidly obese women after bariatric surgery; they demonstrated a tendency toward decreased expression levels from baseline status for CD20 after weight loss (82). Winer et al. demonstrated that B cells and IgG are important pathogenic effectors in the development of obesity-associated insulin resistance and glucose intolerance in diet induced obese mice (83).

The functional associations of CD20 (MS4A1) were explored using the STRING v11 clustering tool (**Figure 4**) (40). CD20 shows strong interactions with the proteins from the immunoglobulin superfamily. These proteins play a role in the B cell receptor signaling pathway. Altered uterine immune profiles in women with repeated implantation failure are undoubtedly present. Some studies confirm the potential importance of the presence of B cells in the pathology of repeated implantation failure/recurrent pregnancy loss  (80, 84). Elevated B cells prior to the Th2 shift, which happens after successful implantation, may operate antagonistically to this process (84).

**Figure 5:**
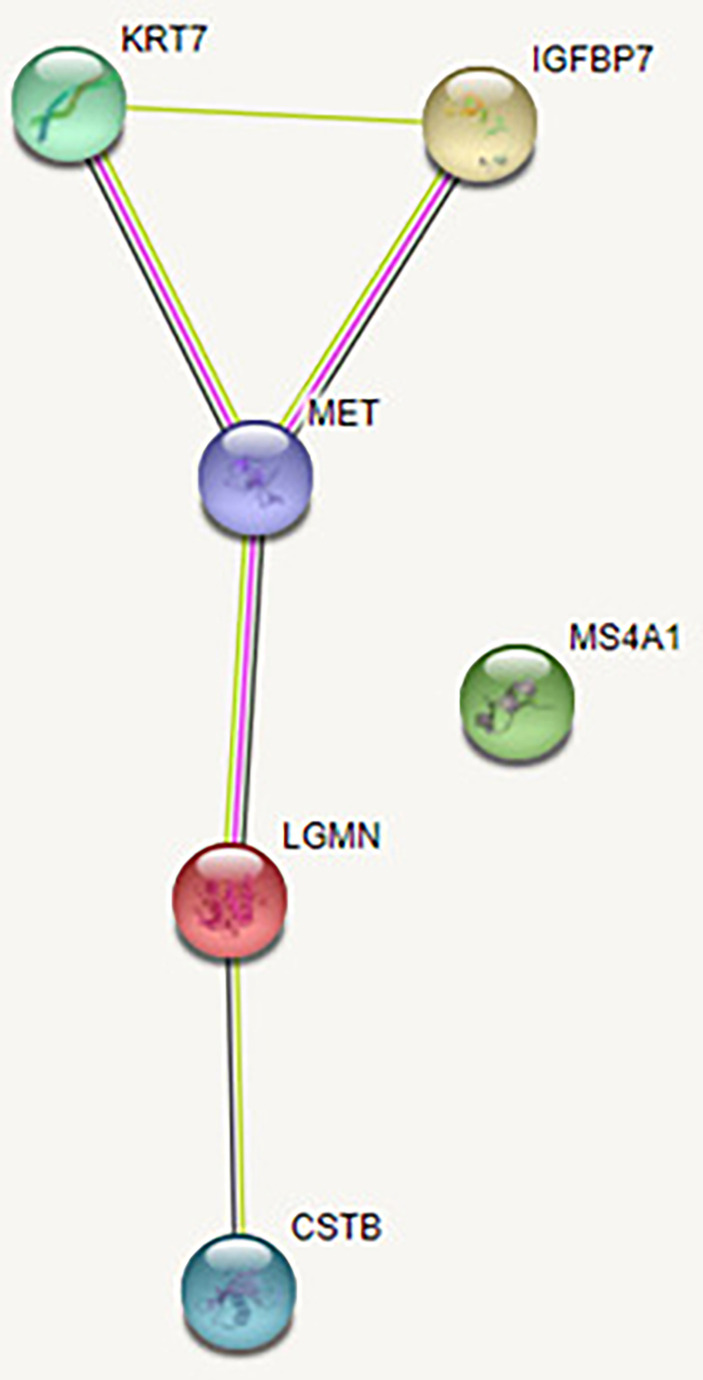
Protein-protein interaction between all investigated proteins. The green line represents the simultaneous mention of related proteins in the published literature. The pink line represents that putative homologs were found interacting in other organisms. The black line represents coexpression of proteins in human or coexpression of their putative homologs in other organisms. Coexpression predicts functional association between the proteins. In our network, there is coexpression between HGF receptor and Legumain and between HGF receptor and CK-7, suggesting their likely significant functional interaction. Legumain (*LGMN*), IGFBP-7 (*IGFBP7*), HGF receptor (*MET*), CK-7 (*KRT7*), Cystatin-B (*CSTB*), CD20 (*MS4A1*).

When investigating interactions between all six studied proteins with high or highest confidence cutoff value, there were no associations between the investigated proteins. After setting the confidence cutoff to low (0.15), an association was found between five proteins with significantly increased abundance at the end of the study. **Figure 5** shows the protein-protein interactions between all investigated proteins, using the STRING v11 clustering tool (40). CD20 protein (the only protein to decline at the end of the study) has no known association with the other five proteins.

### Lifestyle Modifications Reduced the Levels of Fasting Glucose and Free Testosterone and Positively Affected Body Characteristics and Motor Capability Skills

We found significantly lowered fasting blood glucose levels at the end of our intervention. We also observed a statistically significant decrease in free testosterone concentration, at the same time, the average values of FAI index and androstenedione concentration did not change.

A large number of studies have demonstrated metabolic improvements after lifestyle modifications in PCOS patients. Domecq et al. in the systematic review and meta-analysis of nine randomized controlled trials also found a statistically significant effect of lifestyle interventions on fasting blood glucose and insulin level (85). On the contrary, the systematic review by Lim et al., which included eleven studies with reports on fasting glucose or fasting insulin, remained uncertain of the effect of lifestyle intervention on glucose tolerance and fasting glucose (86). Our study assessed insulin resistance using HOMA-IR score. We observed a trend of decreasing fasting insulin concentration and HOMA-IR along with the loss of body weight, although not statistically significant. The systematic review and meta-analysis by Haqq et al. that included 12 studies with 668 participants also reported on HOMA-IR level; they found that HOMA-IR was not significantly different for lifestyle versus usual care groups (87).

In our study, we assessed hyperandrogenism by measuring the concentration of free testosterone, SHBG, FAI, and androstenedione. According to the International evidence-based guideline for the assessment and management of polycystic ovary syndrome, free testosterone measures provide the most optimal accuracy to detect biochemical hyperandrogenism (88). Lim et al. concluded that lifestyle treatment may result in a slightly greater decrease in total testosterone and a greater increase in SHBG and may improve the free androgen index in a recently published Cochrane systematic review (86). Similar findings were published by Haqq et al. (89). Additionally, exercise alone positively affects the elevation of SHBG levels (89). Our study established that the elevation of SHBG concentration was not statistically significant, however, a trend of its increase was observed. A possible reason for the lack of a statistical significance could be the small sample size and the shorter duration of lifestyle interventions. We conclude, therefore, that lifestyle modifications could improve hyperandrogenism in PCOS patients *via* decreased androgen secretion and decreased bioavailability of androgens.

Our study findings confirm the long-standing fact that lifestyle modifications improve several anthropometric markers in PCOS patients and lead to statistically significant changes in body composition and physical capacity. All body characteristics of our participants were statistically significantly improved; at the same time, we also recorded an improvement in all observed motor capabilities. Our findings are consistent with the recently published Cochrane systematic review, which included 15 studies with 498 participants and found greater weight loss, BMI reduction, waist circumference, and waist‐hip ratio reduction with lifestyle treatment compared to minimal treatment (86).

## Conclusions

This study sought to determine how weight loss associated with lifestyle changes (balanced and diverse diets, reduced alcohol and tobacco consumption, regular exercise), affects endometrium specific proteome, endocrine-metabolic characteristics, and motor capabilities of obese women with PCOS and infertility.

Firstly, using the antibody microarrays analysis of endometrial proteome, we discovered an increased protein abundance for Legumain, IGFBP-7, HGF receptor, CK-7, and Cystatin-B in endometrial samples taken at the end of the lifestyle intervention, while CD20 protein abundance was decreased. Some of these proteins have been previously reported in the context of human EM receptivity, whereas the role of others needs to be further explored.

Secondly, we established that lifestyle modifications reduced the levels of fasting glucose and free testosterone and positively affected body characteristics and motor capability skills.

This study may, despite being exploratory in its nature, open up avenues for exploring potentially important biomarkers that may influence endometrial receptivity, both in general and after weight loss.

## Data Availability Statement

The original contributions presented in the study are included in the article/**Supplementary Material**. Further inquiries can be directed to the corresponding author.

## Ethics Statement

The studies involving human participants were reviewed and approved by Medical Ethic Committee of Republic of Slovenia (0120-491-2017). The patients/participants provided their written informed consent to participate in this study.

## Author Contributions

Conceptualization, EBV, TP, MJ and DA; methodology, EBV and MJ; validation, MJ and DA; formal analysis, AS, NJ; investigation, AS, NJ, TP, MJ and DA; resources, AS, TP, NJ, MJ, EBV and DA; data curation, AŠ and DA; writing—original draft preparation, AS, TP and DA; writing—review and editing, EBV, NJ, MJ and DA; visualization, DA; supervision, EBV; project administration, NJ and DA; funding acquisition, EBV. All authors have read and agreed to the published version of the manuscript. All authors are accountable for all aspects of the work in ensuring that questions related to the accuracy or integrity of any part of the work are appropriately investigated and resolved.

## Funding

This research was funded by the Slovenian Research Agency ARRS (Javna agencija za raziskovalno dejavnost Republike Slovenije), grant number J3-8206.

## Conflict of Interest

The authors declare that the research was conducted in the absence of any commercial or financial relationships that could be construed as a potential conflict of interest.

## Publisher’s Note

All claims expressed in this article are solely those of the authors and do not necessarily represent those of their affiliated organizations, or those of the publisher, the editors and the reviewers. Any product that may be evaluated in this article, or claim that may be made by its manufacturer, is not guaranteed or endorsed by the publisher.

## Supplementary Material

Supplementary Table 1 Proteins with differential abundance in END samples and START samples, including proteins that do not reach the significance and logFC thresholds simultaneously. Proteins with a positive logFC value had a higher abundance in END samples, proteins with a negative value in START samples. In addition, p-values adjusted for multiple testing are listed. The Uniprot-Identifier links to the Uniprot-Entry.

Supplementary File S2
**Figure 4**. Functional associations of the studied proteins with all abbreviations.
