# Supplementary material for: The Lifestyle Modifications and Endometrial Proteome Changes of Women With Polycystic Ovary Syndrome and Obesity
Source: Front Endocrinol (Lausanne). 2022 Jun 22;13:888460. doi: 10.3389/fendo.2022.888460 (PMC9258031; doi:10.3389/fendo.2022.888460)
Supplement: Supplementary File S2 — Figure 4 . Functional associations of the studied proteins with all abbreviations. [file Table_2.docx]

Figure 4. Functional associations of the studied proteins. The colored nodes signify a direct interaction with other proteins. The confidence cutoff for showing interaction links has been set to “high” (0.700) for CD20 and ‘highest’ (0.900) for Legumain, IGFBP-7, HGF receptor, CK-7, and Cystatin-B. Legend: **(A)** Legumain (*LGMN*), **(B)** IGFBP-7 (*IGFBP7*), **(C)** HGF receptor (*MET*), **(D)** CK-7 (*KRT7*), **(E)** Cystatin-B (*CSTB*), **(F)** CD20 (*MS4A1*).

(A): Legumain (LGMN), Cubilin (CUBN), Vitamin D-binding protein (GC), Cystatin-M (CST6), Toll-like receptor 7 (TLR7), HLA class II histocompatibility antigen gamma chain (CD74), Major histocompatibility complex, class II, DQ alpha 1 (HLA-DQA1), Major histocompatibility complex, class II, DQ beta 1 (HLA-DQB1), HLA class II histocompatibility antigen, DRB1-15 beta chain (HLA-DRB1), HLA class II histocompatibility antigen, DR beta 5 chain (HLA-DRB5), Major histocompatibility complex, class II, DQ beta 2 (HLA-DQB2).

(B): Insulin-like growth factor-binding protein 7 (IGFBP7), Transcription factor AP-1 (JUN), Interleukin-6 (IL6), Insulin-like growth factor-binding protein 10 (CYR61), Insulin-like growth factor-binding protein 1 (IGFBP1),Insulin-like growth factor-binding protein 3 (IGFBP3), Insulin-like growth factor-binding protein 4 (IGFBP4), Insulin-like growth factor-binding protein (IGFBP5), Cystatin-C (CST3), Fibronectin 1 (FN1), Metalloproteinase inhibitor 1 (TIMP1).

(C): Hepatocyte growth factor receptor (MET), Hepatocyte growth factor (HGF), GTPase HRas (HRAS), GTPase KRas (KRAS), Cadherin-1 (CDH1), Catenin beta-1 (CTNNB1), Growth factor receptor-bound protein 2 (GRB2), E3 ubiquitin-protein ligase CBL (CBL), Signal transducer and activator of transcription 3 (STAT3), SH2-domain containing Phosphatidylinositol-3,4,5-trisphosphate 5-phosphatase 2 (INPPL1), SH3 domain-containing kinase-binding protein 1 (SH3KBP1).

(D) Keratin, type II cytoskeletal 7 (KRT7), Keratin, type I cytoskeletal 9 (KRT9), Keratin, type I cytoskeletal 10 (KRT10), Keratin, type I cytoskeletal 13 (KRT13), Keratin, type I cytoskeletal 14 (KRT14), Keratin, type I cytoskeletal 15 (KRT15), Keratin, type I cytoskeletal 16 (KRT16), Keratin, type I cytoskeletal 17 (KRT17), Keratin, type I cytoskeletal 18 (KRT18), Keratin, type I cytoskeletal 19 (KRT19), Keratin, type I cytoskeletal 20 (KRT20).

(E) Cystatin-B (CSTB), Lactotransferrin (LTF), Haptoglobin-related protein (HP), Spectrin alpha chain, non-erythrocytic 1 (SPTAN1), Beta-2-microglobulin (B2M), Lysozyme C (LYZ), Cathepsin D (CTSD), Cystatin-C (CST3), Cathepsin S (CTSS), Pro-cathepsin H (CTSH), Metalloproteinase inhibitor 2 (TIMP2).

(F) B-lymphocyte antigen CD20 (MS4A1), B-cell antigen receptor complex-associated protein alpha chain (CD79A), B-cell antigen receptor complex-associated protein beta chain (CD79B), B-lymphocyte antigen CD19 (CD19), Membrane-spanning 4-domains subfamily A member 3 (MS4A3), Membrane-spanning 4-domains subfamily A member 7 (MS4A7).
